# Diagnostic Scrutiny and Patterns of Elevated Cancer Risk: Uncovering Overdiagnosis Through Standardized Incidence Ratios

**DOI:** 10.7759/cureus.42439

**Published:** 2023-07-25

**Authors:** Yunchan Chen, Valeria Gutierrez, Luc Morris, Jennifer L Marti

**Affiliations:** 1 Department of Surgery, Weill Cornell Medicine, New York, USA; 2 Head and Neck Service, Memorial Sloan Kettering Cancer Center, New York, USA; 3 Division of Breast Surgical Oncology, Icahn School of Medicine at Mount Sinai, Mount Sinai Hospital, New York, USA

**Keywords:** incidence trends over time, cancer overdiagnosis, cancer epidemiology, cancer incidence rates, cancer screening, cancer prevalence

## Abstract

Certain medical diagnoses and environmental or occupational exposures may be associated with elevated risk of cancer diagnosis, either through causal mechanisms or via increased detection of a subclinical reservoir through increased diagnostic scrutiny (overdiagnosis). The present study aimed to investigate the distribution of elevated cancer risks associated with different diagnoses and exposures. A systematic literature search was conducted to identify studies published in the last 30 years that examined the standardized incidence ratio (SIR) associated with exposures and risk factors. Meta-SIRs for each cancer type were calculated. The distribution of elevated cancer risks was then compared between cancer types previously reported to be susceptible to overdiagnosis and those that have not been associated with overdiagnosis. The review of 108 studies identified four patterns: SIR generally elevated for 1) only overdiagnosis-susceptible cancer types, 2) both overdiagnosed and non-overdiagnosed cancer types, 3) select cancers in accordance with risk factor or exposure, and 4) SIRs that did not exhibit a distinct increase in any cancer type. The distribution of elevated cancer risks may serve as a signature of whether the underlying risk factor or exposure is a carcinogenic process or a mechanism of increased diagnostic scrutiny uncovering clinically occult diseases. The identification of increased cancer risk should be viewed with caution, and analyzing the pattern of elevated cancer risk distribution can potentially reveal conditions that appear to be cancer risk factors but are in fact the result of exposure to medical surveillance or other healthcare activities that lead to the detection of indolent tumors.

## Introduction and background

With the improving quality and utilization of advanced diagnostic technologies, more patients are being diagnosed with cancer, either due to serendipitous detection or organized cancer screening programs [1]. Although this paradigm shift has enabled the identification of certain neoplasms and interceptions with therapies, detecting cancer before the onset of signs and symptoms has both advantages and disadvantages. Cancers grow at vastly different rates and can have significant variability in their potential to cause harm [1,2]. In contrast to rapidly growing cancers, a subset of cancers can exhibit very slow growth and may never become symptomatic or cause morbidity and mortality [1,2]. Some tumors are non-progressive cancers that remain dormant or may even regress, perhaps through the emergence of cellular senescence, immune predation, or outgrowth of vascular and metabolic support [1,3-5].

The epidemiologic term “overdiagnosis,” in the context of cancer, refers to tumors that would not go on to cause any morbidity and mortality if they were never diagnosed [1]. Because “overdiagnosed” tumors pose no risk of harm to a patient, their treatment poses risks without benefits. For example, a patient may undergo surgery, radiation therapy, and/or chemotherapy, as well as psychological distress and financial toxicity. The treatment of overdiagnosed cancers is also a significant source of low-value or no-value care to health systems.

However, because tumors are rarely left untreated, it is not possible to directly categorize individual tumors as overdiagnosed. The assessments of the presence of overdiagnosis have relied on indirect epidemiologic measures. For example, prior studies have examined incidental prostate, thyroid, and breast cancers found at autopsies to confirm the presence of a reservoir of subclinical cancers of these sites [1,6-8]. Other studies have used incidence and mortality trends and lead-time estimates to infer the presence of overdiagnosis [9,10]. Some studies have specifically examined the role of increased diagnostic scrutiny, linking the incidence of certain cancers with the performance of certain diagnostic tests, such as ultrasound exams detecting thyroid cancer, skin biopsies detecting melanoma, and mammograms detecting breast cancer [9,11,12].

To examine the role of diagnostic scrutiny in leading to the detection of cancer more broadly, rather than limited to specific investigational tests, we examined the associations between multiple healthcare conditions and the relative risk of being diagnosed with different types of cancer. We hypothesized that certain medical diagnoses or conditions that lead to increased contact with the healthcare system would increase the incidence of certain types of cancer that are susceptible to overdiagnosis, while not altering the incidence of other types of cancer that are not susceptible to overdiagnosis. By contrast, other conditions that increase the risk of developing cancer would be associated with increased incidence of both categories of cancer.

## Review

Methods

Data Collection

**Figure 1:**
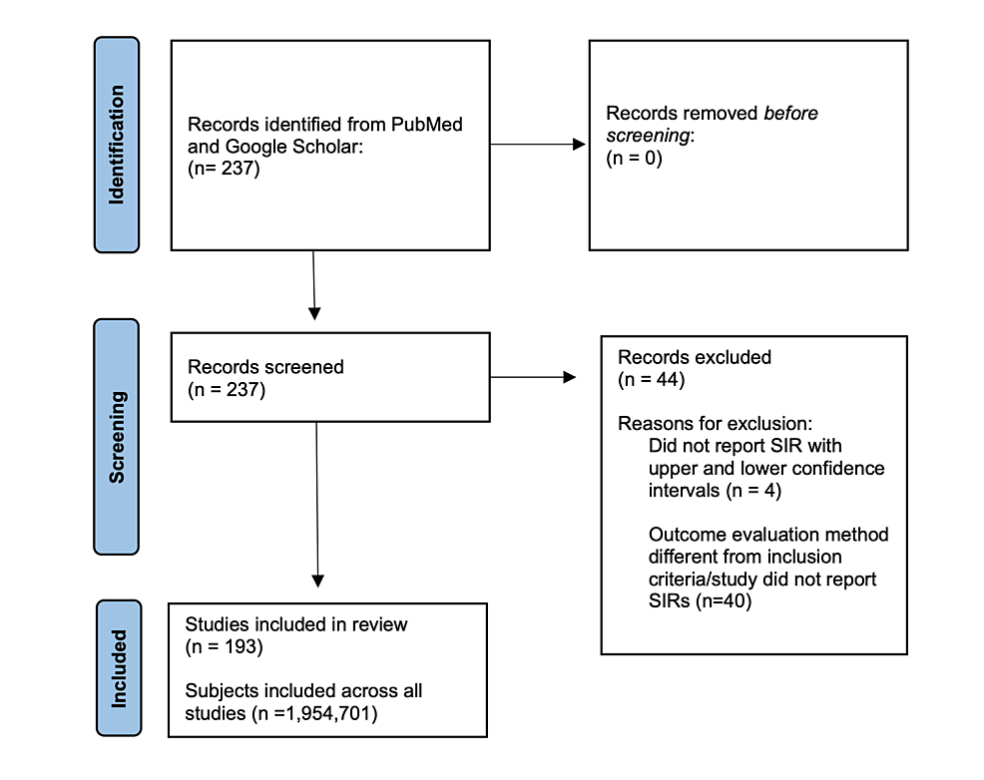
PRISMA diagram PRISMA: Preferred Reporting Items for Systematic Reviews and Meta-Analyses

PubMed and Google Scholar databases were searched for any meta-analyses, systematic reviews, or other studies published between 1991 and 2021 that listed standardized incidence ratios (SIRs) for common cancer types, including thyroid, breast, melanoma, prostate, lung, gastric, head and neck, colorectal, pancreatic, and liver cancers, and associated conditions, illnesses, exposures, or general characteristics. Search terms included “Thyroid cancer AND SIR/Standardized incidence ratio,” “Breast Cancer AND SIR/Standardized incidence ratio,” “Melanoma and SIR/Standardized incidence ratio,” “Prostate Cancer AND SIR/Standardized incidence ratio,” “Lung cancer AND SIR/Standardized incidence ratio,” “Gastric Cancer AND SIR/Standardized incidence ratio,” “Head and Neck Cancer AND SIR/Standardized incidence ratio,” “Colorectal Cancer AND SIR/Standardized incidence ratio,” “Pancreatic Cancer AND SIR/Standardized incidence ratio,” “Liver Cancer AND SIR/Standardized incidence ratio,” and “Cancers AND SIR/Standardized incidence ratio.” All SIRs and confidence intervals reported for any type of cancer were included. Studies that reported only an SIR without a 95% confidence interval or did not report SIRs were excluded. In total, 237 studies were screened, of which 193 studies consisting of 1,954,701 subjects met the inclusion criteria (Figure 1). Studies were reviewed by two researchers and grouped by thematic categories. The review question used to frame the study can be seen in Appendix 1. 

Statistical Analysis

The SIR, defined as the number of observed cancer cases divided by the number of expected cases, is a measure that compares the incidence of a disease to a reference population, allowing for the consideration of covariates, such as age, gender, geographic distribution, and other demographic features [13]. To evaluate the aggregate data for the cancers and exposure/risk factor groups, we developed a method to estimate composite SIRs across studies within a category.

To perform a fixed-effects model meta-analysis, the inverse variance statistical method was used to create a weighted average of the effect sizes. If subgroup count data were not provided, the standard error (SE) associated with each SIR was estimated from the given 95% confidence interval. For SIRs, we used the same approach as odds ratio SE approximation, where SE = $\frac{ln(\text{upper 95}\% \text{ CI})-ln(\text{lower 95}\% \text{ CI})}{2*1.96}$. The extended derivation for the method can be found in Appendix 2.

Once SIRs and SEs were identified and grouped into risk factor categories, a risk factor-cancer composite SIR was calculated using the inverse-variance weighted average $\frac{\sum y_i(1/SE_{i})^{2}}{(1/SE_{i})^{2}}$, where $y_i$ represents $SIR_i$ and $SE_i$ represents the SE of the estimate. Aggregations were performed with a meta-analysis software (Cochrane Review Manager version 5.4, The Cochrane Collaboration, United Kingdom).

Results

After a systematic review, 108 studies fulfilled the inclusion criteria. A total of 22 categories were identified from the risk factors and exposures, excluding categories with fewer than five relevant studies (Table 1). Cancer types were categorized as either overdiagnosis-susceptible or generally non-overdiagnosed, based on prior literature: cancer types with consistent literature evidence for a subset being susceptible to overdiagnosis (“overdiagnosis-susceptible”) were thyroid, prostate, breast, melanoma, kidney, and non-small cell lung cancers [1,9]. Other cancer types lacking evidence for overdiagnosis were categorized as non-overdiagnosed.

**Table 1:** Categories by exposure and risk factors ACE: angiotensin-converting enzyme; NSAIDs: non-steroidal anti-inflammatory drugs; HIV/AIDS: human immunodeficiency virus/acquired immunodeficiency syndrome; HCV: hepatitis C virus; HBV: hepatitis B virus; HSV: herpes simplex virus; PTEN: phosphatase and tensin homolog; BRCA 1 & 2: breast cancer 1 & 2; HNPCC: hereditary non-polyposis colorectal cancer; CT: computed tomography; WTC: World Trade Center; COPD: chronic obstructive pulmonary disease

Category	Condition description in the manuscript	Number of studies
Organ transplants	Liver transplants, kidney transplants, heart transplants, childhood transplant recipients, solid organ transplants	10
Occupations without specific hazardous agent exposure	Public safety workers, farmers, airline pilot, cabin crew, female flight attendants, priest, nurses, meat workers	8
Medication usage	Amiodarone, proton pump inhibitors, diethylstilbestrol, antibiotics, ACE inhibitors, aspirin/NSAIDs	6
Adult therapeutic radiation history	Total body irradiation, nasopharyngeal radiation therapy, cervical spine disorders treated with radiation, uterine cancer (irradiated), prostate cancer (irradiated), thyroid cancer primary (irradiated) (radioactive iodine), breast cancer (irradiated, treatment with combined 252Cf neutron brachytherapy with external beam radiotherapy)	9
Occupational radiation exposure	Medical workers exposed to low-dose ionizing radiation, radiation workers/radiologic technologists, Chernobyl clean-up workers, Korean radiation workers, Los Alamos National Laboratory workers, uranium underground miners	7
Occupations with toxic exposures	Pesticide applicators/spouses, propylene manufacturers, polycyclic aromatic hydrocarbon exposure, hazardous waste exposure, workers at petrochemical plants, rubber manufacturing industry, polycyclic aromatic hydrocarbon exposure/aluminum plant workers, semi-conductor workers, spinning weaving unit (exposed to bleaching, scouring, and dyeing agents and other chemical compounds, e.g., formaldehyde, phenol, different inorganic acids, and aromatic amines (benzene)), dyeing-finishing unit, firefighters	10
Family history of cancer/terminal condition	First-degree relatives with papillary thyroid cancer, at least one first-degree relative with breast carcinoma in situ, family members of patients with cystic fibrosis, first-degree relatives with borderline or in situ prostatic neoplasia, women under 40 with family history of breast cancer, parent with breast cancer, parent with ovarian cancer, relatives of children with germ cell tumors	7
Gastroenterology conditions	Gallstones without cholecystectomy, non-alcoholic chronic pancreatitis, colorectal adenoma, rectosigmoid adenomas, ulcers (gastric, duodenal), celiac disease, ulcerative colitis, Crohn’s disease, cholecystectomy, appendectomy in childhood	13
Infectious diseases	HIV/AIDS, HCV, HBV	4
Gynecological conditions	Endometriosis, benign solid ovarian tumor, placental abruption, women evaluated for infertility, women taking clomiphene, women who had assisted reproduction, women with hysterectomies, women using levonorgestrel-releasing intrauterine system, placental abruption, HSV gynecological infection	8
Rheumatological conditions	Sarcoidosis, systemic sclerosis, Sjogren’s, ankylosing spondylitis, rheumatoid arthritis, severe psoriasis, systemic lupus erythematosus, inflammatory bowel disease	15
Thyroid disease	Hyper/hypothyroidism, toxic adenoma, Grave’s disease, thyrotoxicosis, acromegaly, male infertility	8
Neurology/psychiatric conditions	Schizophrenia, Parkinson’s, myotonic dystrophy, intellectual disability, meningioma, cervical/spinal disorder	7
Alcohol history/cirrhosis	Alcoholism, liver cirrhosis, chronic pancreatitis	5
Adult cancer history	Prior adulthood cancer diagnoses include non-thyroid malignancy, testicular cancer, primary male breast cancer, lymphoma, melanoma, colorectal cancer, prostate cancer, breast cancer, soft tissue cancer, cutaneous melanoma, oral cancer, families with adult onset sarcoma, lung cancer, chronic lymphocytic leukemia, prior prostate cancer diagnosis, urothelial cancer in men, cervical cancer, endometrial cancer, colorectal cancer, lymphoma, ovarian cancer, pancreatic cancer, basal cell carcinoma, squamous cell carcinoma, primary thyroid cancer (metachronous), cholangiocarcinoma (metachronous), primary breast cancer (synchronous)	25
Hereditary cancer mutation carrier	PTEN, BRCA 1 & 2, Ras, Noonan syndrome, HNPCC mutation	8
Obesity	-	6
Immigrant	Immigrants to Sweden, Germans resettling in Tomsk Russia, re-settlers from former Soviet Union	3
Diabetes	Type 1 and 2 diabetes, diabetic nephropathy	15
Childhood therapeutic radiation	Childhood exposure to radiation oncology and CT scans	8
World Trade Center (WTC) exposure	Population includes WTC workers, general responders, firefighters, rescue/recovery workers, and police force	6
Smoking	Smokers, smokers with vascular diseases, smokers with COPD	5

Exposures were categorized into four patterns based on the comparison of SIRs in overdiagnosis-susceptible vs. non-overdiagnosed cancer types.

**Figure 2:**
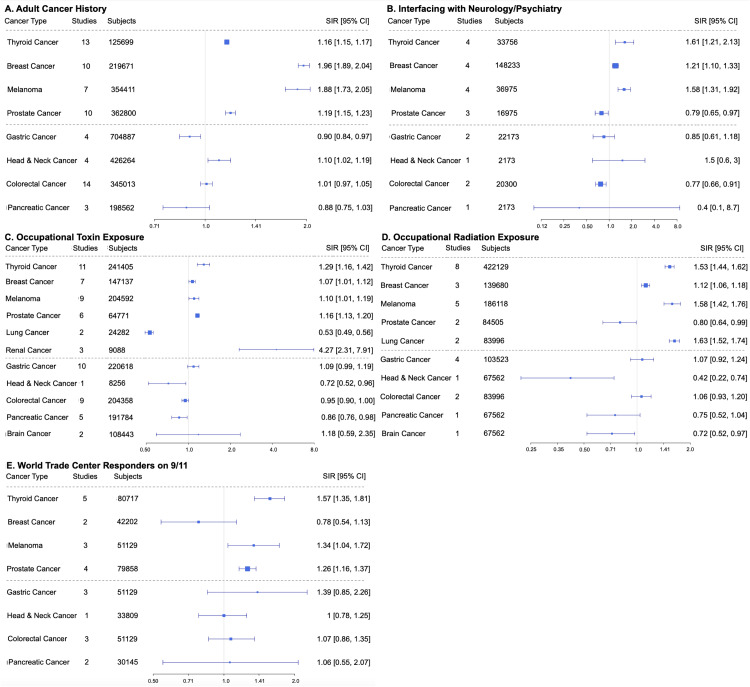
Pattern 1 risk factors in order (left to right, top to bottom): A) adult cancer history, B) interface with neurology or psychiatry, C) occupational toxin exposure, D) occupational radiation exposure, and E) World Trade Center (WTC) responders on 9/11. SIR: standardized incidence ratio; CI: confidence interval

In Pattern 1, or the “increased diagnostic scrutiny” pattern, exposures/conditions led to SIR >1 only in overdiagnosis-susceptible cancers, whereas non-overdiagnosed cancers had SIRs ≤1. Exposures/conditions in Pattern 1 include adult cancer history, interface with neurology or psychiatry, occupational toxin exposure, occupational radiation exposure, and World Trade Center (WTC) 9/11 responders (Figure 2).

**Figure 3:**
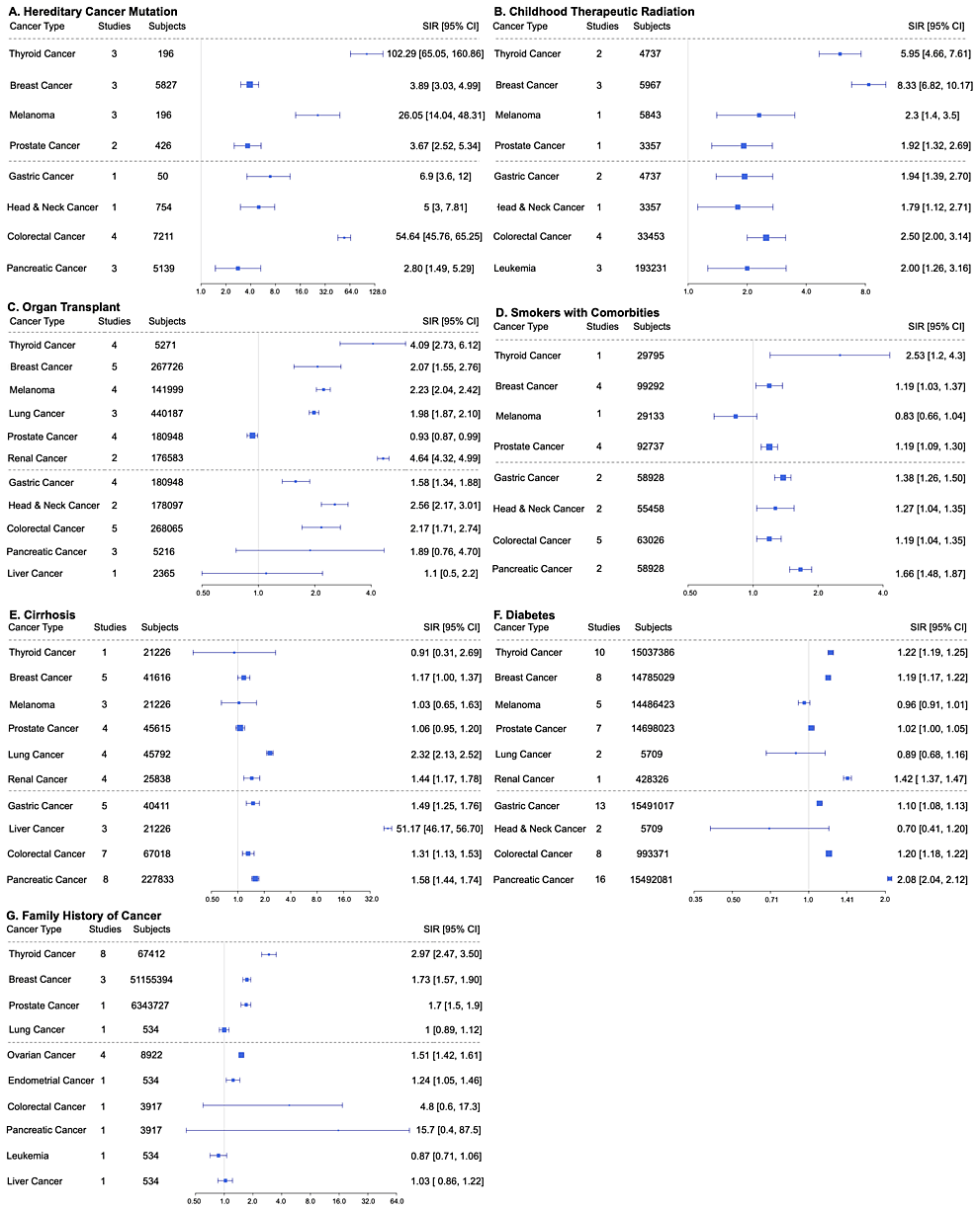
Pattern 2 risk factors in order (left to right, top to bottom): A) hereditary cancer mutation carriers, B) childhood therapeutic radiation, C) organ transplants, D) smokers with comorbidities, E) cirrhosis, F) diabetes, and G) family history of cancer. SIR: standardized incidence ratio; CI: confidence interval

In Pattern 2, or “broadly elevated cancer risk,” most malignancies with statistically significant SIRs had values above 1. Examples of Pattern 2 include hereditary cancer mutation carriers, childhood therapeutic radiation, solid organ transplant recipients, smokers with comorbidities, cirrhosis, diabetes, and family history of cancer (Figure 3).

**Figure 4:**
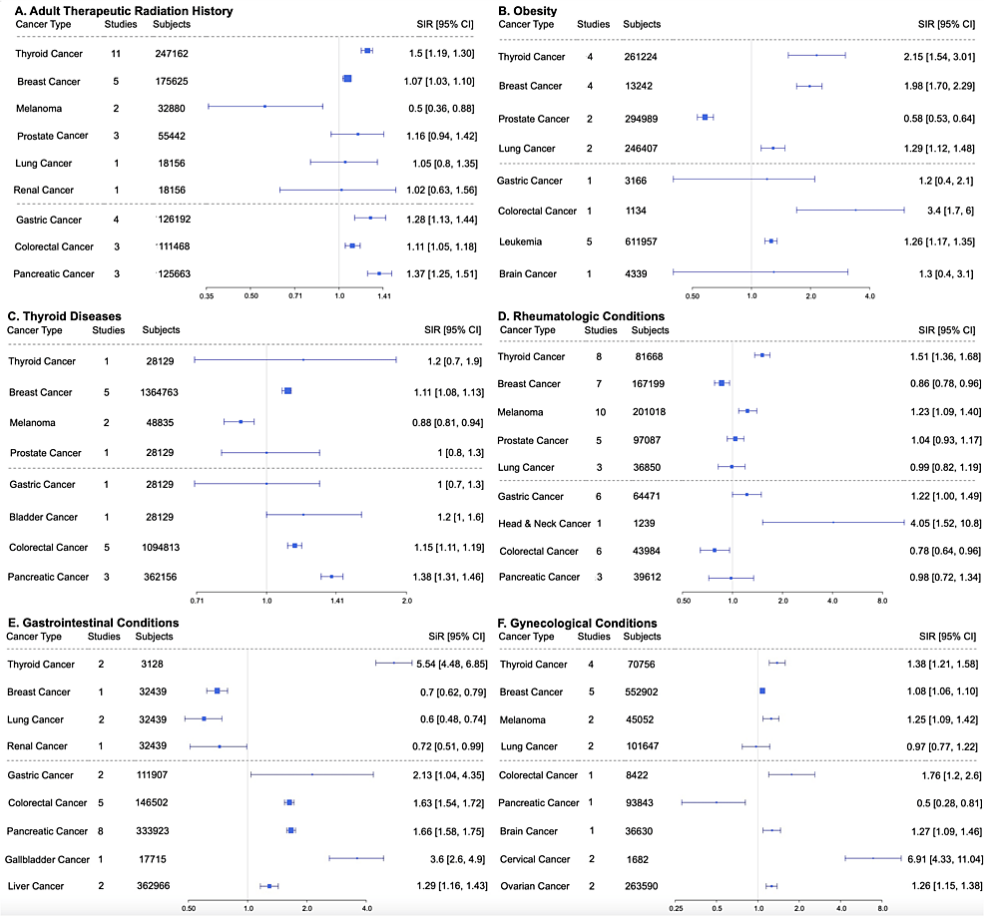
Pattern 3 risk factors in order (left to right, top to bottom): A) adult therapeutic radiation history, B) obesity, C) thyroid diseases, D) rheumatologic conditions, E) gastrointestinal conditions, and F) gynecological conditions. SIR: standardized incidence ratio; CI: confidence interval

In Pattern 3, or “cancer-specific risk,” only a limited number of cancer types had SIRs >1. Risk factors include adult therapeutic radiation history (thyroid and breast cancer), obesity (several cancer types), thyroid diseases (thyroid cancer), rheumatologic conditions (non-Hodgkin's lymphoma, lung cancer, melanoma, kidney cancer, thyroid cancer, and leukemia), gastrointestinal conditions (esophageal cancer, gastrointestinal (GI) cancer, and hepatocellular carcinoma), and gynecological conditions (ovarian cancer, endometrial cancer, cervical cancer, vaginal cancer, and breast cancer) (Figure 4) [14-21].

**Figure 5:**
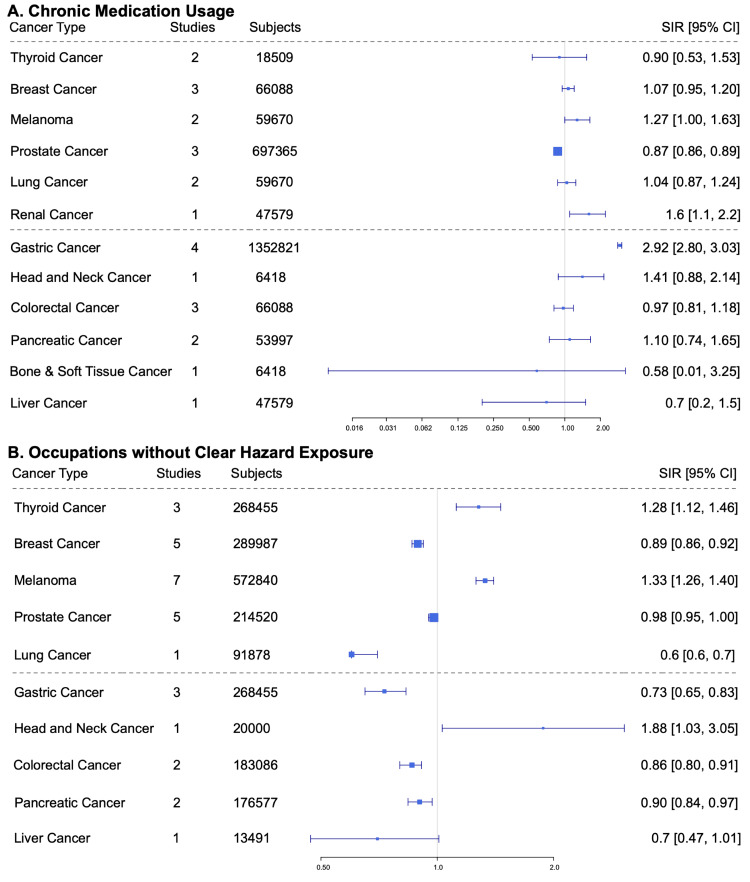
Pattern 4 risk factors in order (left to right): A) chronic medication usage and B) occupations without clear hazardous exposure. SIR: standardized incidence ratio; CI: confidence interval

In Pattern 4, no discernable incidence increase was observed. Categories fitting this pattern were chronic medication usage and occupations without clear hazardous exposure (Figure 5).

Discussion

Diagnostic scrutiny, i.e., the intensity of examination and testing that a patient undergoes, is a natural and usually well-intentioned result of contact with the healthcare system. Benign and malignant tumors are examples of the many medical conditions that can sometimes be clinically occult and pose no future harm to a patient’s health, but which are discovered incidentally as a result of diagnostic examinations or tests. This association, which can result in overdiagnosis, is difficult to study quantitatively. Here, we examine the degree to which different medical exposures and conditions affect the subsequent risk of being diagnosed with several types of cancer - considering both cancer types that are susceptible to overdiagnosis and cancer types in which overdiagnosis does not occur. To our knowledge, the association between diagnostic scrutiny and cancer diagnosis has not been examined in a comparative manner across cancer types. In this study, we leverage the phenomenon of diagnostic scrutiny to describe the strength of association between certain risk factors, exposures and medical conditions, and the subsequent diagnosis of cancer.

By leveraging different types of exposures and risk factors that patients face, we find that some of these exposures only increase the risk of developing certain types of cancer; in some cases, specific cancer types for which prior data implicates a causal linkage to the exposure. In other cases, however, risks are elevated across the group of overdiagnosis-susceptible cancer types, but not in other cancer types, consistent with increased detection due to higher diagnostic scrutiny.

Assessing the link between diagnostic scrutiny and cancer diagnosis in a quantitative fashion has been challenging, prompting researchers to employ various inferential techniques. For example, some studies have analyzed tissues obtained during autopsies to assess the prevalence of a subclinical reservoir of occult cancer in patients lacking a cancer history. These studies have discovered that occult breast, thyroid, and prostate cancers can be detected at autopsy in patients without any relevant signs or symptoms and that the prevalence of these cancers increases with age, suggesting that there are potentially large subclinical reservoirs of certain types of cancer in healthy individuals [1,22]. Other studies have compared the incidence of early to advanced cancers at the time of detection, and trends over time as diagnostic technologies become more widespread. In the presence of overdiagnosis, a rise in cancer incidence would not result in a rise in cancer mortality rates [1]. The results of these studies are supportive of the existence of overdiagnosis in certain cancer types, but they do not generate a quantitative estimate of how strong the association between diagnostic scrutiny and the increased detection of certain cancers may be.

To complement these prior approaches, in the present study, we investigate elevated cancer risks resulting from different medical exposures and conditions. Some carcinogenic exposures increase the risk of developing one or more types of cancer, while other factors associated with diagnostic scrutiny will only increase the risk of cancer types in which overdiagnosis has been shown to occur, i.e., cancers with a sizable subclinical reservoir, in which increased diagnostic testing can easily lead to more detected cancers.

We found that exposures that led patients to have increased contact with the healthcare system, receive closer monitoring, or interface more frequently with physicians generally lead to more diagnoses of overdiagnosis-susceptible cancer types, including prostate cancer, thyroid cancer, breast cancer, and melanoma. These findings are consistent with prior studies identifying associations between county-level income levels and the number of newly diagnosed cases of prostate cancer, thyroid cancer, breast cancer, and melanoma [23]. Similar data have linked regional levels of healthcare access, education, and income to higher numbers of diagnosed thyroid cancers, melanoma, breast cancers, and prostate cancers [24-29].

Many of the exposures or diagnoses in this study have been previously linked with the utilization of healthcare or other contact with the healthcare system. For example, a study evaluating screening practices revealed significantly higher rates of diagnostic tests and compliance with self-exams among cancer survivors than individuals without similar medical history [30]. In addition, past literature has shown significantly increased healthcare utilization and costs, hospitalizations, primary care visits, laboratory tests, and radiology services among patients with complex psychiatric conditions and neurological disorders [31-35]. Workers with hazardous waste or toxin exposure may also receive greater medical surveillance or biological monitoring as a part of routine workplace safety and contamination assessment [36,37]. Occupational radiation exposure can lead to escalated diagnostic scrutiny and result in an elevated number of subclinical “incidentalomas” [24]. The World Trade Center Health Program (WTCHP), established in an effort to provide 9/11 responders and survivors with medical surveillance and treatment for a panel of chronic conditions, may similarly increase patient contact with the healthcare system and diagnoses of other medical conditions [38].

The link between diagnostic scrutiny and increased detection of overdiagnosis-susceptible cancers is shown in Pattern 1. An important caveat to such an analysis is that these data do not provide causal evidence but rather evidence in support of prior data of overdiagnosis. However, meta-SIRs may provide useful insights as estimates of the effect size associated with increased healthcare utilization. Furthermore, these findings do not rule out the possibility of coexistent true increases in cancer risk associated with these exposures.

By contrast, Pattern 2 risk factors demonstrated higher incidence rates for multiple malignancies, both overdiagnosis-susceptible and non-overdiagnosed, due to known cancer risk factors, such as certain inherited germline mutations, radiation exposure, immunosuppression, and smoking. For example, pathogenic germline mutations in oncogenes, tumor suppressor genes, DNA repair genes, and other cell cycle regulators have been shown to confer susceptibility to cancer development [39-41]. An additional cancer risk factor is ionizing radiation, particularly in younger individuals [42,43]. In utero or childhood exposure to radiation significantly increases the odds ratio for cancer-related mortality [43]. Solid organ transplant recipients are also at higher risk of developing a wide range of malignancies due to profound immunosuppression and potential oncologic viral infections [44]. Meta-analyses evaluating modifiable risk factors found cigarette smoking and alcohol consumption to be among the leading causes of cancer cases and deaths [45]. Diabetes has been cited as a potential cause of tumorigenesis due to the hyperglycemic and inflammatory environment it creates. Family history of cancer is also associated with a concordant increase in individual risks due to shared genetic factors and lifestyle aspects and common behaviors [46,47]. For all of these risk factors, cancer SIRs were elevated for most malignancies, indicating the oncogenic effects of these exposures. 

In Pattern 3, risk factors were linked only to a limited number of cancer types, suggestive of (but not definitively proving) a causal link between that exposure and the development of specific cancer types. In some of these cases, there is consistent biological or epidemiological data to support a possible link. For example, thyroid diseases and rheumatological conditions are known to predispose patients to chronic, systemic inflammation, leading to the accumulation of reactive oxygen species and subsequent DNA damage, an established mechanism for the neoplastic transformation of normal cells [48]. Obesity was found to be associated with a relative increase in the incidence of most types of cancers except prostate cancer [49]. Adult therapeutic radiation also led to an increase in most cancer incidences except prostate, but to a lower degree than childhood radiation. GI and gynecological conditions led to elevated cancer incidences in their respective organ systems. Based on these results, for Pattern 3, we are unable to make definitive conclusions, other than to surmise that there is not clear evidence for these risk factors serving as either general carcinogenic risk factors (more consistent with Pattern 2) or drivers of diagnostic scrutiny and overdiagnosis (more consistent with Pattern 1).

Finally, the risk factors categorized as Pattern 4 did not consistently show associations with escalated risks of malignancy or displayed mixed signals. In these cases, no definitive conclusions can be drawn.

## Conclusions

Analyzing the pattern of elevated cancer risk distribution can support prior evidence of overdiagnosis and provide initial point estimates of the strength of association between certain medical conditions that may, through elevated diagnostic scrutiny, increase the detection rates of indolent tumors.

As research increasingly shed light on this topic, a re-examination of the current infrastructure surrounding cancer screening and diagnosis may be warranted. Healthcare authorities may undertake a thorough review of existing screening guidelines to strike a balance between the benefits of early detection and the potential harms of overdiagnosis, which could involve reassessing the targeted age groups and recommended frequency of tests. Policymakers may prioritize informed decision-making for patients and advocate for effective communication strategies. Furthermore, optimizing screening resource allocation may enhance coverage for higher-risk groups. Ultimately, continual monitoring and adaptation of policies and practices will be essential to improve patient outcomes. 

The future direction of this study includes evaluating the signatures of cancer overdiagnosis in correlation to resource availability and sociodemographic factors.

There are a number of important limitations to this study. Observational SIR rates were analyzed in an exploratory and hypothesis-generating format. These findings do not establish causality, nor do they clearly prove the presence of overdiagnosis. However, they do provide point estimates for the degree to which certain exposures or diagnoses increase the detection rate of certain overdiagnosis-prone cancers. In the context of prior literature using other inferential techniques to estimate the presence of overdiagnosis, these data provide useful estimates of the strength of association with diagnostic scrutiny. Ultimately, the data for the exposures in Pattern 1 are most useful as additional supportive data for other studies of diagnostic scrutiny, overdetection, and overdiagnosis.

## Figures and Tables

**Figure 6:**
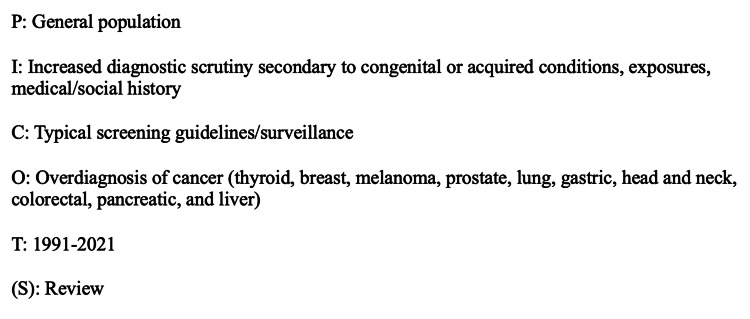
PICOT(S) used in the present study (Patient, Intervention, Comparison, Outcome, Timing, Setting).

**Figure 7:**
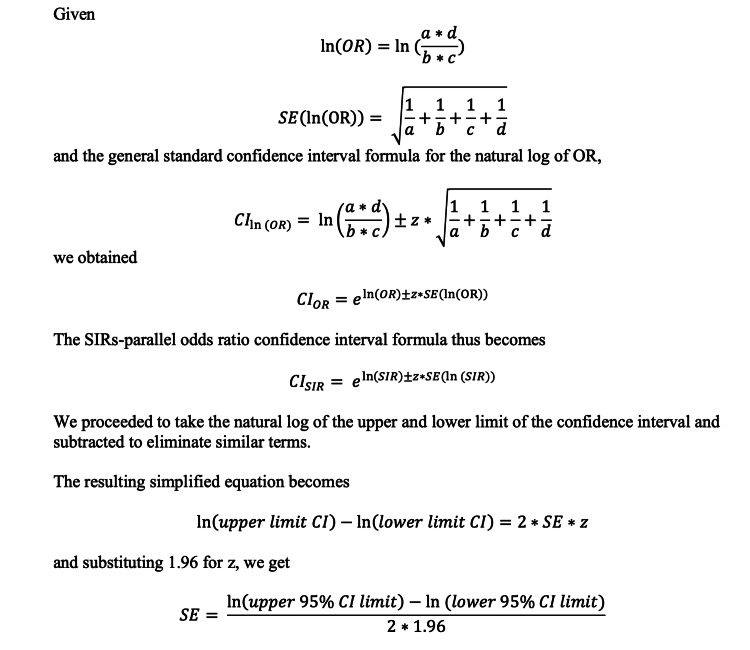
Expanded calculations.

## Appendices

Appendix 1 

Appendix 2 
